# A partial economic evaluation of blended learning in teaching health research methods: a three-university collaboration in South Africa, Sweden, and Uganda

**DOI:** 10.3402/gha.v9.28058

**Published:** 2016-10-06

**Authors:** Minna Kumpu, Salla Atkins, Merrick Zwarenstein, Lungiswa Nkonki, for the ARCADE consortium

**Affiliations:** 1 Department of Public Health Sciences, Global Health (IHCAR), Karolinska Institutet, Stockholm, Sweden; 2 Centre for Studies in Family Medicine, Department of Family Medicine, Western University, London, Ontario, Canada; 3 Centre for Health Systems and Services Research, Faculty of Medicine and Health Sciences, Stellenbosch University, Stellenbosch, South Africa

**Keywords:** cost analysis, education, public health professional, students, public health, blended learning, health systems research

## Abstract

**Background:**

Novel research training approaches are needed in global health, particularly in sub-Saharan African universities, to support strengthening of health systems and services. Blended learning (BL), combining face-to-face teaching with computer-based technologies, is also an accessible and flexible education method for teaching global health and related topics. When organised as inter-institutional collaboration, BL also has potential for sharing teaching resources. However, there is insufficient data on the costs of BL in higher education.

**Objective:**

Our goal was to evaluate the total provider costs of BL in teaching health research methods in a three-university collaboration.

**Design:**

A retrospective evaluation was performed on a BL course on randomised controlled trials, which was led by Stellenbosch University (SU) in South Africa and joined by Swedish and Ugandan universities. For all three universities, the costs of the BL course were evaluated using activity-based costing with an ingredients approach. For SU, the costs of the same course delivered with a classroom learning (CL) approach were also estimated. The learning outcomes of both approaches were explored using course grades as an intermediate outcome measure.

**Results:**

In this contextually bound pilot evaluation, BL had substantially higher costs than the traditional CL approach in South Africa, even when average per-site or per-student costs were considered. Staff costs were the major cost driver in both approaches, but total staff costs were three times higher for the BL course at SU. This implies that inter-institutional BL can be more time consuming, for example, due to use of new technologies. Explorative findings indicated that there was little difference in students’ learning outcomes.

**Conclusions:**

The total provider costs of the inter-institutional BL course were higher than the CL course at SU. Long-term economic evaluations of BL with societal perspective are warranted before conclusions on full costs and consequences of BL in teaching global health topics can be made.

## Introduction

Global health needs strong health systems (1). To this end, policymakers need reliable, relevant, and strong evidence on costs and effectiveness of interventions to support decision-making, particularly from health systems research. The current skills gap in health systems research (2) resulted in some Millennium Development Goals not being achieved (3). Therefore, long-term investments at the individual, institutional, and national levels are needed to build health research capacity (4). Without this effort in health research capacity building, achieving the Sustainable Development Goals by 2030 will be challenging.

The African Capacity Development on Health Systems and Services Research (ARCADE HSSR) project focused on building research capacity in African countries (5). The project took a blended learning (BL) approach as a way to build individual capacity, combined with other approaches targeted at the institutional level. BL is one possible approach for increasing local training of health professionals in resource-constrained settings (6), and thus it can contribute to global health efforts. BL refers to a teaching approach that combines face-to-face classroom learning (CL) and instruction utilising computer-based technologies (7, 8), often involving reduction in classroom teaching hours (9–11). ARCADE HSSR focused particularly on collaborative course delivery across northern and southern institutions (12). Such inter-institutional BL can improve the accessibility of education by reducing travel and potentially save costs as teaching resources are shared. The use of video/audio conferencing technology enabling discussion across sites is close to traditional face-to-face teaching in terms of interactivity (6), with the added benefits of flexibility and a diverse participant group.

Reviews on BL (6, 13, 14) have concluded that it has the potential to improve learner engagement through online assessments (14), enhance meaningful learning experiences (13), and increase faculty efficiency (6). Systematic reviews and meta-analyses have concluded that BL learning outcomes are similar to other teaching methods (15–17). However, BL also presents significant demands on institutions in terms of infrastructure and staff skills, which may not always be readily available (6, 18) and which can require costly investments in technologies (19, 20) and increase faculty workload (21). In contrast, implementation of BL can also lead to cost reductions, such as due to decreased need for physical infrastructure and improved scheduling (22).

BL is increasingly popular (6, 23), but limited data are available on its total costs compared to CL in higher education (6). These data can help to assess the potential of BL to support health research training and thus health system capacity building, especially in resource-constrained settings. We aimed to address this gap by evaluating the total provider costs of BL when organised as a three-university collaboration between South Africa, Sweden, and Uganda. The secondary aim was to compare the costs of BL to CL and to explore differences in learning outcomes in South Africa.

## Methods

One of the first courses delivered using BL approaches in ARCADE HSSR was a course on randomised controlled trials (RCTs). The course was taught using CL at SU from 2009 as part of the master of science in epidemiology programme (24). The shift towards BL began in 2011 and starting in 2012 the course was delivered as BL and as an inter-institutional collaboration. It was organised by Stellenbosch University (SU) in South Africa, with Karolinska Institutet (KI) in Sweden and Makerere University (MU) in Uganda contributing students and tutors as participating universities.

This retrospective economic evaluation was conducted to estimate the total provider costs of BL in teaching a course on RCTs as a three-university collaboration in the ARCADE HSSR framework. A cost description was performed for all three institutions – SU, KI, and MU. In addition, at SU, a CL course was included in the evaluation as an historical comparator. The learning outcomes of BL were also explored and compared to those of CL at SU, using course grades as an intermediate outcome measure. As both the BL and CL versions of the course had the same learning content and objectives, course grades were a good source of outcome data available for the retrospective evaluation. The students’ final grades on both the BL and CL courses were formed based on assignments (50%: three assignments in the CL course and four in the BL course) and a final exam (50%). The grading scale on the course was 0–100 and the pass mark was 50% (50), which is the standard on postgraduate-level courses.

### RCT course organisation

In terms of running the courses, the final year of running each course was evaluated (2010 and 2013 for the CL and BL courses, respectively). Both the CL and the BL courses had the same learning objectives, and the same themes were covered in teaching (design and different types of RCTs, practical issues related to conducting trials, statistical methods used in data analysis). Descriptive statistics on both courses are presented in Table 1. MU followed the SU course outline and schedule closely. At KI, there were some differences, as total course hours (determined by the number of study credits students received) were one-third of the hours specified for SU and MU. Therefore the final exam was not included in the course and 2 hours less of classroom teaching was included, as one scheduled session was organised separately for KI students as a shorter version (Table 1).

**Table 1:** Participant numbers and content of courses on randomised controlled trials delivered as BL in 2013 with inter-institutional collaboration between SU, MU, and KI, and as CL in 2010 at SU

	RCT BL (2013)			RCT CL (2010)
		
	SU	MU	KI	SU
General course information				
ECTS	Na	na	1.5	na
Learning time required, h	120	120	40	120
Classroom learning				
Time scheduled, h	24	24	22	40
Occasions, *n*	4	4	4	7
Online learning				
Time estimated, h	88^a^	88^a^	18^b^	na
Self-study				
Time estimated, h	Na	na	na	80
Evaluations				
Assignments, *n*	4	4	4	2
Final exam included	Yes	yes	no	yes
Forming of final grade				
Weight of assignments,%	50	50	100	50
Weight of final exam,%	50	50	na	50
Enrolments				
Accepted to course, *n*	20	5	9	13
Started the course, *n*	16	4	8	13
Dropped-out during course, *n*	–	–	–	–

BL, blended learning course; CL, classroom learning course; ECTS, European Credit Transfer System; KI, Karolinska Institutet; MU, Makerere University; RCT, randomised controlled trial; SU, Stellenbosch University; na, not applicable.

a Hours specified in course outline; does not add up to total hours of learning required together with classroom hours as breaks were also counted as classroom hours in course schedule.

b Not specified in course outline; calculated as difference between total and classroom hours.

The BL course included 16 hours less of scheduled CL time compared to the CL course at SU (Table 1). The BL course included eight self-study online sessions, which included readings, videos, and self-assessment quizzes that were provided through the online learning platform Moodle (www.moodle.org). SU used a classroom with videoconferencing equipment for teaching the course, and KI used a meeting room with similar functionality. Students from MU participated in the teaching sessions in a classroom using their own laptops, because of low student numbers and non-availability of videoconferencing equipment for such a small number of students. The course also required a bridge for videoconferencing to connect all sites and allow for interaction. The sites used Microsoft Lync software to connect through the bridge. The faculty used Camtasia software in 2013 to make videos of the teaching content, which were made available for students to watch online.

### Costing approach

All costs were examined from the providers’ perspective, using activity-based costing. Using activity-based costing, all activities needed to produce the course were first specified, after which cost ingredients were categorised for each activity. An ingredients approach was used to collect data on resources used in order to identify all necessary inputs regardless of the funding source. This approach consisted of identifying and valuing all resources required to set up and run the BL and CL courses and calculating the total costs for both approaches. Resource use was tracked from the start of the project and course planning until the end of 2013. All inputs were tracked by and linked to the site that incurred the cost, even if the purchase or work input benefitted all sites to some extent. This illustrated the needs of institutions with different roles, KI having the overall managerial role of ARCADE HSSR, SU representing the course organiser, and MU having a smaller participant role in this BL activity.

### Data collection methods

We conducted a document review to achieve an overview of activities related to BL in the ARCADE HSSR project, by examining the project's grant agreement (5). The activities identified were used as probes in the semi-structured key informant interviews. We selected key informants purposively to include key staff, such as the ARCADE HSSR project initiator, coordinator, and assistant; the SU principal investigator; teachers of the BL and CL courses at SU; and local tutors for the BL course at KI and SU (one person with a dual role of project coordinator and tutor). We asked all interviewees to estimate inputs related to face-to-face meetings, person time, physical spaces, other infrastructure, and other inputs. The interviews were semi-structured, as they were primarily designed to identify all relevant inputs and to measure these in appropriate physical units, such as hours of work. The secondary aim of the interviews was to gain further understanding of the BL activities in ARCADE HSSR and collect information on BL and CL courses, to ensure that all relevant cost-incurring activities were taken into consideration in the evaluation.

### Collection and valuation of inputs

An overview of the collection and valuation of inputs is presented in Table 2.

**Table 2:** Collection and valuation of inputs required to start up and run the ARCADE HSSR project and the RCT courses as BL and CL

	Data source for inputs		Valuation of inputs			
				
	Document review	Semi-structured interviews	Financial records	Estimation based on average market prices	Start-up or running costs of project or course	Additional information
Staff time	Identification of activities used for resource allocation	Time input in different phases, years the work took place and activities the time was used on	Salary data for people paid through the ARCADE HSSR project	Salaries for people not paid through the project (based on the expertise and country)	Allocated partly to all, based on key informant estimations.	Benefits outside the monthly salary were excluded
Meetings	Attendees and length of meetings from meeting minutes	Identification of required face-to-face meetings	All travel costs (transport, accommodation, per diems) and part of organising costs (e.g. meals)	Organising costs not retrievable from records (e.g. venue hire)	Start-up costs of the project or course, depending on the meeting	Full or partial costs included, depending on the role of the participant
Space (teaching)	Teaching hours from course outlines	Description of required teaching facilities		Rent costs (required size, location and equipment)	Course running costs (BL and CL)	Staff use of office space excluded in this evaluation
Equipment		Identification of required equipment	For equipment that were specifically purchased for the use of BL activities in the ARCADE HSSR	Items needed on the courses but were not paid through the project	Full costs of the items included in the start-up costs of the project	Staff use of basic office equipment (e.g. staff computers, printers) excluded
Licences		Identification of required licences	Majority of licence costs	Licences that were provided for free for testing in the project, or were purchased by the university for other purpose	Annual licences used on the course included in running costs of BL course. Licences only tested in the project considered start-up costs of the project.	

ARCADE HSSR, African Regional Capacity Development for Health Systems and Services Research; BL, blended learning course; CL, classroom learning, RCT, randomised contolled trial.

For participants who were identified by key informants for their time contribution to the project or courses, time input to meetings was not calculated separately to avoid double-counting, and only travel costs retrievable from the project expenses (e.g. transport, accommodation, and per diems) were included as meeting-specific costs. Only hours used to participate in the meeting were included for those meeting participants that were not otherwise included in staff time. This time was valued based on professional positions. These participants contributed to the meeting content, but as none of the key informants had mentioned them as key for the project or courses, we considered the inclusion of full costs excessive.

The cost of videoconferencing equipment was covered by approximating the cost of the spaces that had the necessary equipment, and this cost was included in the running cost of BL. SU had purchased the bridge for the videoconferencing system, which was included in the start-up costs of BL in ARCADE HSSR. As Microsoft Lync was not specifically purchased for the project, the initial once-off licence fee was not costed, but the yearly fee for three devices was included as an approximation of a recurring cost of using the program in running the course. Several other communication cooperation systems were tested in ARCADE HSSR (Skype Premium, Adobe Connect, MiniSip) at the project level, thus the cost for 1 year's use of each of these programmes is included in the ARCADE HSSR start-up costs of the BL course. The BL start-up costs also included an once-off licence fee for the Camtasia software. Other purchases in ARCADE HSSR that participants considered necessary for starting up BL included two computers, two microphones, and a webcam, which were all purchased at KI. The annualisation and depreciation of these costs are discussed at the end of this section.

The valuation of the inputs was done by expenditure reviews of project financial records. For those BL resources not included in project records and resources needed in the CL version of the course, national market prices were used to approximate costs. A common overhead is added to instances of outside purchasing of services from the university in the main study setting of South Africa. This figure, 10%, was used as the overhead cost on the final sum, to account for resources that serve several departments or programmes (e.g. costs of general university administration, cleaning, electricity, and heating of the buildings).

Costs were collected in local currencies where this information was available (KI and part of SU costs). Costs from MU and part of the SU costs were collected from EU reporting, where all costs were presented in euros. All currencies were first converted to US dollars using the average exchange rate of the year when the cost had taken place (25). Costs were adjusted to the prices of the chosen base year, 2013, using a consumer price index (CPI) specific for each country (26–28).

All equipment costs were classified as capital costs, as the useful life of all the items was more than 1 year, and start-up costs were considered capital expenditure. Annualisation was undertaken by using 2013 interest rates for each country's 10-year government bonds as the discount rate (29–31) and 5 years as the useful life of capital inputs (information and communication technology [ICT] items and start-up costs), by consulting the standard table for the appropriate annualisation factor (32).

### Allocation of inputs

Work related to BL in general, done as part of the ARCADE HSSR project, was vital for achieving the knowledge, acquiring the equipment, and formulating the materials needed to deliver the RCT course as BL. Thus, interviewees were also asked about inputs needed for planning of the ARCADE HSSR project as a whole and on general BL activities within the project, as well as what share of these inputs was relevant to the RCT course. The averages of these estimations were used to allocate part of the general project inputs to the RCT BL course. Key informant estimations were also used to allocate staff hours related to BL activities in the ARCADE HSSR project to start-up and operational needs. As start-up inputs will benefit BL activities at the institutions and the courses for many years, these costs were annualised, and the annual cost for 1 year was included in the final costs. As the BL course had been running for 3 years, one-third of the ARCADE HSSR project costs allocated to running the RCT course were included in the costs of running the 2013 RCT BL course.

### Categorisation of inputs

Three input categories were formed to present the collected data under both start-up and running costs. Planning and management included inputs related to the course and project planning and management; central activities, such as general ARCADE HSSR meetings; and coordination and administrative work. The ICT capacity category included all inputs needed for ICT capacity building and maintenance. The course development and delivery consisted of inputs needed for the course module review and development, as well as course delivery. Space costs included only teaching space costs, as meeting space costs were included in the meetings category.

### Sensitivity analysis

A one-way sensitivity analysis was conducted to assess the role of possible uncertainties in the data. Staff costs were identified as the key driver of total cost and also as most subject to recall bias; thus the total cost of time inputs was varied by 20% up and down. As space costs vary considerably depending on location and limited data were available for evaluation of space costs with videoconferencing equipment, space costs were varied by 20% up and down. Meeting costs were very specific to the project under evaluation (e.g. the number of meetings needed and number of people travelling); thus they were varied on a larger scale, 50% up and down. Furthermore, as significant variation existed in estimations of what proportion of costs related to BL activities in ARCADE HSSR project should be allocated to the RCT BL course, this parameter was varied to represent the lowest and highest estimations.

## Results

### Descriptive statistics of students enrolled in the RCT courses

Thirteen master's students participated in the RCT course that was delivered as CL at SU in 2010 (Table 1). Altogether 28 students participated in the RCT course using the BL approach in 2013, with 57% of students at SU, 29% at KI, and 14% at MU. Participants were PhD-level students at KI, students who had just finished their master's degree at MU, and master's students at SU. For both courses, 31% of students were women. All students in both courses passed the course.

### Learning outcomes

Explorative findings on course grades suggest that there was little difference between the groups in learning outcomes. The mean final grade of students taking the BL and CL courses was 67.3 (SD 7.6) and 64.3 (SD 5.8), respectively.

### Costs

The economic costs of the RCT courses are presented as start-up and running costs and are summarised under three main activities (Table 3). The total provider costs of BL, as implemented in the ARCADE HSSR project, for delivering a course on RCTs as a three-university collaboration was USD 68,000. Of these costs, 36% were related to start-up costs and 64% to running the course one time. The total costs at SU were USD 29,476 for BL and USD 13,699 for CL.

**Table 3:** Economic costs (in US dollars) of courses on RCTs delivered as BL in 2013 with inter-institutional collaboration between SU, MU, and KI and as CL in 2010 at SU

	RCT BL (2013)								RCT CL (2010)	
			
	SU		MU		KI		Total		SU	
						
	USD^a^	%	USD^a^	%	USD^a^	%	USD^a^	%	USD^a^	%
Start-up costs										
Planning and management										
Staff	1,508		1,174		2,041		4,723		499	
Meetings	1,022		2,047		1,058		4,128		–	
ICT capacity										
Staff	1,733		89		168		1,990		–	
Equipment and licences	2,617		–		632		3,249		–	
Course development and delivery										
Staff	1,722		122		244		2,088		2,483	
Meetings	252		37		7,703		7,992		–	
Total start-up costs	8,855	30	3,469	19	11,846	58	24,171	36	2,982	22
Running costs										
Planning and management										
Staff	3,674		1,443		2,898		8,015		5,322	
ICT capacity										
Staff	5,131		165		350		5,646		–	
Equipment and licences	102		–		–		102		–	
Course development and delivery										
Staff	8,116		12,270		2,244		22,630		2,082	
Teaching space	3,598		888		3,010		7,496		3,314	
Total running costs	20,621	70	14,766	81	8,501	42	43,888	64	10,718	78
Total costs	29,476	100	18,235	100	20,347	100	68,059	100	13,699	100
Average cost per student	1,842		4,559		2,543		2,431		1,054	

BL, blended learning course; CL, classroom learning course; ICT, information and communication technology; KI, Karolinska Institutet; MU, Makerere University; RCT, randomised contolled trial; SU, Stellenbosch University.

a Costs from other years than 2013 adjusted to prices of 2013, using a consumer price index (CPI) specific for each country (27–29).

Staff costs were the major cost category within most activities and in total costs (Table 3). For the BL course, of start-up, running, and total costs, 36, 83, and 66% were staff costs, respectively. In the CL course, all start-up costs, 69% of running costs, and 76% of total costs were staff costs.

In the BL course's start-up costs, meetings were the most significant source of costs, representing 50% of the total start-up costs. The source of these costs was five international meetings that were relevant for starting up the BL activities within the project. Three of these were general ARCADE HSSR project meetings, where only part of the content was related to BL and therefore only part of the total meeting costs were allocated to BL, according to interviewee estimates (50% for one meeting and 25% for two meetings). One international meeting was solely focused on BL, and one focused directly on transforming the RCT course to BL.

Of the start-up costs of the BL course, 13% were equipment and licence costs, whereas such costs were less than 1% of running costs. Teaching space costs represented 17 and 31% of running costs of the BL and CL courses, respectively. The average per-site cost of the RCT BL course was USD 22,700, for example decreasing the SU costs by 23% when compared to the site-specific cost.

At SU, the costs of starting up and running the RCT course as BL were 115% higher than starting up and running the course as CL (Table 4). Of the incremental cost, 63% was from start-up and 37% from running the course. Staff costs accounted for 73% of the incremental cost. The major factor behind the higher staff costs in BL was more staff working hours within all studied activities. Despite the lower number of classroom hours on the BL course, the staff hours needed, for example, for running the course at SU were over double the hours estimated for running the CL course. As the number of students in the compared courses differed by three students, incremental costs per student were also analysed. Per-student costs of BL were 75% higher compared to CL. When the average per-site costs were examined, the incremental costs for SU came down by 43%, to USD 8,987, but the costs of BL still remained 66% higher compared to CL.

**Table 4:** Incremental costs and effectiveness of a course on RCTs delivered as BL in 2013 compared to delivery as CL in 2010 at SU

	RCT BL, SU (2013) compared to RCT CL, SU (2010)
Total incremental costs,^a^ USD^b^	15,776
Start-up	5,873
Running	9,903
Per-student incremental costs,^a^ USD^b^	788
Start-up	324
Running	464

BL, blended learning course; CL, classroom learning course; RCT, randomised contolled trial; SU, Stellenbosch University.

a Costs of BL course minus costs of CL course at SU.

b Costs from other years than 2013 have been adjusted to prices of 2013, using a consumer price index (CPI) specific for each country (27–29).

Varying the estimates of the allocation of ARCADE HSSR costs to the RCT BL course had the highest impact on total costs in the sensitivity analysis (Table 5). Allocating project costs to the RCT course according to the highest allocation estimate increased the total costs of RCT BL course by 25%. Variation in meeting and space costs had the smallest impact on the total course costs.

**Table 5:** Sensitivity analysis of economic costs of courses on RCTs delivered as BL in 2013 with inter-institutional collaboration between SU, MU, and KI and as CL in 2010 at SU

	RCT BL (2013)				RCT CL (2010)
		
	SU	MU	KI	Total	SU
Baseline cost, USD^1^	29,476	18,235	20,347	68,059	13,699
Change (%) in baseline cost					
Staff costs +20%	+15%	+17%	+8%	+13%	+15%
Staff costs −20%	−15%	−17%	−8%	−13%	−15%
Highest allocation of ARCADE HSSR costs to RCT course	+33%	+18%	+19%	+25%	na
Lowest allocation of ARCADE HSSR costs to RCT course	−17%	−10%	−10%	−13%	na
Meeting costs +50%	+2%	+6%	+22%	+9%	na
Meeting costs −50%	−2%	−6%	−22%	−9%	na
Space costs +20%	+2%	+1%	+3%	+2%	+5%
Space costs −20%	−2%	−1%	−3%	−2%	−5%

BL, blended learning course; CL, classroom learning course; KI, Karolinska Institutet; MU, Makerere University; RCT, randomised contolled trial; SU, Stellenbosch University; na, not applicable.

a Costs from other years than 2013 have been adjusted to prices of 2013, using a consumer price index (CPI) specific for each country (27–29).

## Discussion

Our research indicated that delivering the course as BL incurred more than double the costs that were estimated for the CL format of the course at the leading university, SU, which also had the highest number of participating students. However, costs remained higher even after evening out the costs of BL between participating universities and after considering the per-student costs. Staff costs seemed to drive the total cost for both approaches, similarly to other studies (33). No notable difference was found in students’ learning outcomes, as also identified in other studies (6, 15–17).

Even though the BL course included fewer classroom hours, the results show that staff hours for course development and delivery were significantly higher for the BL course than the CL course. This finding was also highlighted by lecturers involved in developing and delivering BL courses in the ARCADE projects (18). This result was unsurprising, as others have suggested BL may lead to increased faculty workload due to the need to create online content and learn new technologies (21). In terms of staff hours it is also important to note that different people were involved in the delivery of the BL and CL courses, which in itself is a source of variation in terms of time use, salaries, and also evaluation of time inputs. Despite these initial high inputs, which should be taken into account by institutions embarking on BL approaches, it is very likely that in future iterations of the BL courses staff will be more familiar with the content and technology and thus spend less time in delivering the course. However, if ARCADE HSSR's approach of real-time connections between sites for teaching and discussions is continued, it is likely that space costs will remain high. The higher per-hour costs of teaching spaces with videoconferencing capability nulls the savings potential of the fewer teaching hours reported elsewhere for BL (22). In contrast, in future meeting costs could decrease, as collaborations between universities become older, staff more experienced, and the need for international meetings decreases.

In the present evaluation we were able to both compare costs and explore one outcome of BL and CL for only one institution. Furthermore, our comparator (CL) at one institution was an historical control. There is a need for rigorous evaluation studies, such as RCTs, with a comparable time frame and outcomes that have a broader spectrum of the hypothesised benefits of BL – expanding the coverage of teaching health systems research and enhancing student learning. Before these types of rigorous evaluations are available, no firm conclusions should be made on cost-effectiveness of BL compared to CL.

The findings of this evaluation are very context-specific, presenting an example of the costs of applying BL as an inter-institutional collaboration between three sites. As this evaluation included only the providers’ perspective, no conclusions can be made on the full societal costs of BL, as for example students’ costs were not included. This evaluation included the first BL course offered in the ARCADE HSSR project, and even if the third year of running the course was looked at in terms of the running costs, the findings represent the transition phase from CL to BL. Interviewees often highlighted that setting up and running the more recent BL courses implemented within ARCADE HSSR has been easier. Nonetheless, the present evaluation provides a full cost description of offering a BL course, including all inputs needed to start-up and run the courses, and not just actual purchases by a specific project, and it can thus be used as preliminary data on provider cost structures related to the shift from CL to BL in teaching health research methods to masters-level students as an inter-institutional collaboration.

This pilot-scale evaluation offers new insight into the total provider costs of BL as organised in an inter-institutional collaboration with a multicountry setting. Intuitively, such efforts could have a great advantage in global health education, in bringing lecturers and students together from different settings (34), thus possibly resulting in new innovations and creative approaches to solving global health problems (35). In fact, the overall results of the ARCADE project are promising (36), with satisfied lecturers (18), satisfied students (37), and the overall advantage of building capacity in low- and middle-income settings in research skills related to global health (12). However, cost is a key concern when implementing any educational programme (38). This concern raises the question of whether approaches using BL are the answer to the research capacity shortages in low and middle-income countries (LMICs). Are there better (and cheaper) ways of building capacity, would for example sandwich training approaches (39) result in increased numbers of skilled researchers in LMICs? These approaches, while designed to mitigate brain drain by building facilities for practising skills at the home institution (40), might still have a greater risk for brain drain than blended education (41). Further research is therefore needed to determine whether BL courses truly do become less time- and cost-intensive in the long term, as well as on alternative approaches to capacity building in LMICs.

## Conclusions

This contextually bound pilot economic evaluation focused on costing an inter-institutional BL course on RCTs, a key skill in health systems and global health research. The evaluation demonstrated that BL had substantially higher costs than the traditional CL approach in South Africa. Despite these costs, BL, especially when implemented with the intention of sharing limited teaching resources between institutions and increasing accessibility of education, has the potential to support global health research capacity building in resource-constrained settings. Further work investigating the long-term costs of BL, including societal perspectives and analysis of consequences, as well as strategies for cost reduction are warranted to determine the true cost-effectiveness of BL compared to traditional teaching approaches. Further exploration of capacity building approaches in LMICs are also needed.
